# Healthy Minds Index: A brief measure of the core dimensions of well-being

**DOI:** 10.1371/journal.pone.0299352

**Published:** 2024-05-10

**Authors:** Tammi R. A. Kral, Pelin Kesebir, Liz Redford, Cortland J. Dahl, Christine D. Wilson-Mendenhall, Matthew J. Hirshberg, Richard J. Davidson, Raquel Tatar

**Affiliations:** 1 Healthy Minds Innovations, Madison, Wisconsin, United States of America; 2 Center for Healthy Minds, University of Wisconsin–Madison, Madison, Wisconsin, United States of America; 3 Department of Psychology, University of Wisconsin–Madison, Madison, Wisconsin, United States of America; De Montfort University, UNITED KINGDOM

## Abstract

We developed a self-report measure of psychological well-being for teens and adults, the Healthy Minds Index, based on a novel theory that four trainable pillars underlie well-being: awareness, connection, insight, and purpose. Ninety-seven items were developed and revised by experts and guided by qualitative testing with teens (n = 32; average age = 16.0 years). After assessing the internal validity and factor structure in teens (n = 1607; average age = 16.7 years) and adults (n = 420; average age = 45.6 years), we reduced the survey to 17 items. We then validated the factor structure, internal and convergent and divergent validity, and retest reliability of the 17-item Healthy Minds Index in two new teen samples (study 1: n = 1492, average age = 15.7 years; study 2: n = 295, average age = 16.1 years), and one adult sample (n = 285; average age = 45.3 years). The Healthy Minds Index demonstrated adequate validity and provided a comprehensive measure of a novel theory of psychological well-being that includes two domains not found in other conceptualizations of this construct—awareness and insight. This measure will be invaluable for primary research on well-being and as a translational tool to assess the impact and efficacy of widely used behavioral training programs on these core dimensions of wellbeing.

## Introduction

On both the individual and societal level, human flourishing is a highly desirable goal. Flourish is defined as “to grow or develop successfully” in the Cambridge English Dictionary, and as synonymous with “thrive” and “prosper” in the Meriam Webster Dictionary. The latter dictionary defines well-being similarly, as “the state of being happy, healthy, or prosperous”. Various lines of research attest to the possibility of deliberately cultivating psychological well-being. However, a unifying framework that clarifies the dimensions of well-being that can be cultivated through training had not been introduced until recently. Integrating evidence from well-being research, cognitive, affective and contemplative neuroscience, and clinical psychology, Dahl, Wilson-Mendenhall and Davidson [1] put forth such a framework. This framework comprises four core dimensions, which have been robustly linked to well-being: awareness, connection, insight, and purpose.

We sought to validate a novel measure of flourishing based on Dahl et al.’s [1] framework for well-being, in teens (i.e., ages 14–18) and adults (i.e., >18 years old), that aligns with areas of skills development that are central to flourishing and often the focus of wellness training: awareness, connection, insight, and purpose. The present work builds from prior conceptions of well-being, including Ryff and Keyes’ Psychological Well-Being index, which includes the domains of purpose in life and positive relations with others [2]. The self-report measure of well-being developed in the present study characterizes the additional domains of awareness and insight, which do not appear in prior conceptions or measures of well-being, and which are necessary to adequately capture the full range of processes that contribute to well-being in a single measure.

This new well-being framework arrives in the context of a crisis in well-being among teens [3] and adults [4]. Particularly in teens, very little focus has emerged specifically on the measurement of well-being. A consistent measure of well-being across development may allow deeper insight into the emergence of the core dimensions of well-being and the way these dimensions of well-being are associated with positive outcomes across the lifetime, starting in early adolescence. Therefore, the main goal of the present work was to develop a psychometrically valid, reliable, and easily implementable self-report measure to capture how teens and adults vary on these four core dimensions of well-being. We will refer to these dimensions collectively as the Healthy Minds Framework.

## The four dimensions of the Healthy Minds framework

### Awareness

In the Healthy Minds framework, awareness refers to heightened attentiveness to the external cues in the environment, as well as to internal cues such as bodily sensations, thoughts, and feelings. People at the high end of this dimension are typically aware of what they are doing, who they are with, and their own internal states. People on the low end, on the other hand, are easily distracted and frequently find themselves acting on “autopilot”.

An important component of awareness is meta-awareness. Meta-awareness refers to an awareness of the processes of conscious experience as they occur in real time. For instance, when we recognize an emotion inside us (e.g., anger) before it leads to a reaction, or when we suddenly realize that we had been lost in thought, these are examples of meta-awareness [5, 6]. The qualities of attentiveness and awareness have been closely linked to healthy psychological functioning [7, 8].

### Connection

Connection refers to a benevolent orientation toward other people that promotes healthy relationships and positive social interactions. It encompasses positive social perceptions (e.g., gratitude, trust, appreciation) as well as a desire and a sense of responsibility for the well-being of others—even those who are outside of one’s immediate social circles. People on the high end of this dimension generally have warm social interactions, think well of and wish well for others, and are willing to balance others’ best interests with their own in their decision-making. People on the low end, on the other hand, are more cynical toward others, have more selfish motivations and less positive social interactions. Various aspects of the connection dimension have been robustly linked to greater well-being [9, 10].

### Insight

Insight, in the Healthy Minds framework, refers to an ongoing awareness of how one’s internal psychological processes (e.g., emotions, thoughts, beliefs, memories) influence one’s subjective experience of both the internal and external world. People on the high end of this dimension can recognize the impact of their own thoughts and emotions on how they feel and how they act. Those on the low end, on the other hand, lack the intuitive access into their psychological processes and cannot use that information to their advantage. Greater levels of insight have been associated with greater levels of well-being [11], whereas low levels of insight are considered to be a hallmark of psychological disorders [12].

### Purpose

Purpose refers to a sense of clarity regarding what is important in one’s life and how one wants to live. People on the high end of the purpose dimension have clear values and personally meaningful aims that guide their day-to-day living. People on the low end of this dimension, on the other hand, perceive little significance in their pursuits and are uncertain about what makes their life worth living. They lack goals and aspirations that structure their life and provide it with an overarching narrative. Research has linked a sense of purpose and meaning in life consistently to well-being [13, 14].

### Overview of studies

Table 1 provides an overview of the methodological approach to validating the Healthy Minds Index (HMx). The HMx scale items were generated and revised based on a combination of expert input, user experience (UX) interviews and a series of 4 studies with teens. Then the validity and reliability of the HMx was assessed across 4 additional studies, in both teen and adult samples. Across these studies, we examined factor structure, internal consistency, convergent and divergent validity, and test-retest reliability of the HMx. To succinctly present the results, we have organized the results by psychometric analysis, and thus present and discuss the studies relevant to a specific psychometric validation goal together (e.g., item generation, convergent and divergent validity).

**Table 1:** Summary of Healthy Minds Index validation studies in order of occurrence.

Study name	N	Objectives
Qualitative Interviews	32	Gather teens’ input on clarity of items and scale language
Scale Development	1607, total	Factor analysis & scale revision, separately for the 4 dimensions of the Healthy Minds Framework (with about n = 400, each)
(Teen Study D)		
Adult Study 1	420	Full validation of revised HMx (online; Qualtrics)
Teen Study 1	1492;	Full validation and test-retest with 3-month interval (in-person)
	934 at retest	
Teen Study 2	285;	Convergent & divergent validity, internal consistency, and test-retest with 2-week interval (online; Qualtrics)
	81 at retest	
Adult Study 2	281;	Internal consistency and test-retest with 2-week interval (online; Prolific)
	96 at retest	

## Methods

### Participants

In all studies, participants were either adults (>18 years old) or teens 13–18 years old. Participants for the qualitative, scale development interviews were recruited from the Madison, WI community using flyers, Craigslist ads, and school district mailing lists; through Facebook posts; and through the mailing list of the Center for Healthy Minds (via e-mail). Participants for the validation studies were recruited through an online survey platform (Qualtrics or Prolific), or through the Character Lab Research Network (CLRN), to complete an online survey on tools to measure well-being. Participants for the retest studies were recruited from those who completed the first survey in the corresponding validation study, and for the online samples, the retest studies were capped at 100 participants, based on a combination of logistical constraints and a power analysis. Demographic information for participants in each study is shown in Table 2. All adult participants provided written consent and minors provided written assent in a digital consent form, and this study was approved by the Advarra Institutional Review Board (IRB), protocol number Pro00033991. The IRB waived the requirement for parental consent of minors, as the study was deemed no more than minimal risk to participants. Participants in the UX testing were compensated with gift cards, and participants in online samples were compensated according to the practices of the corresponding recruitment organization (Qualtrics or Prolific participant panels). Recruitment and data collection began in September 2019 for the UX research and ended with adult study 2 in April 2022.

**Table 2:** Summary of study demographics.

Study name	Genders					Mean age, years (SD^f^)	Age Min, Max	Race & Ethnicity								
								White	Black	East Asian	South Asian	Native American/ Aboriginal	Latino	Native Hawaiian/ Pacific Islander	Other	NA
	F^a^	M^b^	N^c^	O^d^	NA^e^											
UX	17	3	0	0	12	16.0 (1.2)	14, 18	10	0	3	0	0	1	0	0	18
Teen D	760	817	26	4	0	16.7 (1.1)	14, 17	967	252	48	42	14	259	9	16	0
Teen 1	635	648	17	10	182	15.7 (1.2)	13, 18	441	204	53	475	11	11	63	52	182
Teen 2	159	113	10	3	0	16.0 (1.4)	14, 18	118	48	15	13	7	57	2	25	0
Adult 1	250	167	2	1	0	45.6 (17.6)	18, 85	201	70	19	9	9	84	8	20	0
Adult 2	142	137	2	0	0	45.3 (16.5)	18, 92	188	41	19	4	3	10	1	5	0

^a^F = female

^b^M = male

^c^N = nonbinary

^d^O = other/ prefer not to answer

^e^NA = no answer/ no data

^f^SD = standard deviation

### Inclusion and exclusion criteria

Inclusion criteria were the ability to speak and read English and residing in the United States of America. Participants in the adult studies had to be 18 years of age or older, and participants in the teen studies had to be between the ages of 13 and 18 years old. For studies conducted through the Character Lab Research Network, sample sizes were determined based on convenience sampling used by the network. In all other studies, studies were powered to detect small to medium effect sizes, with 80% power to detect an effect at *p* <0.05.

All data were checked for straight-line responses, which were not present in any of the datasets. Data collected from online panels were further inspected and excluded for response times averaging under 315 ms per word, to remove “speeder” participants who may have sped through the surveys without reading the questions. This threshold has been used previously as a proxy for the minimum duration required to read and cognitively process a survey question [15], and resulted in exclusion of data from 10 participants from teen study 2. Data from adult study 2 (online) were further excluded for failure of the attention check (n = 4 excluded).

### Item generation & scale development

Content experts generated and iteratively reviewed items for each of the Healthy Minds Framework dimensions. The original scale had 97 items, and the initial expert review reduced it to 80 total items. The following guidelines were used for decisions on removing versus retaining items during each round of expert review: 1) maintaining a mix of “easy”, “mid”, and “hard” questions per domain (i.e., most participants expected to score high on “easy” items and low on “hard” items); 2) avoiding reverse-coded items; 3) meet Protection of Pupil Rights Amendment (PPRA) standards (https://www2.ed.gov/policy/gen/guid/fpco/ppra/parents.html); and 4) avoiding socially desirable or evaluative language.

We then conducted a series of qualitative, user experience (UX) interviews with 32 teens to assess and revise the scales for each domain. Participants in the UX studies completed a virtual video interview in which they read each item aloud, for each of the scales of the HMx and said aloud what came to mind. Interviewers then followed up with questions to understand whether the questions in the scales were clear, and that participants understood the items as intended. For example, interviewers asked, “What are you thinking as you look at this?” and “Can you take me through the steps of how you came to that answer?” The qualitative insights from the UX interviews were used to adapt the language of individual items, and to guide expert review in subsequent revisions.

In Teen Study “D”, we then conducted a set of factor analyses to assess the scale construction for each of the four dimensions of well-being, and to further revise the scale to remove poorly performing items, while retaining the minimal number of items sufficient for validity. This study consisted of 4 sub-studies (i–iv), in separate samples, to assess scales for each of the 4 domains: Awareness (i), Connection (ii), Insight (iii), and Purpose (iv). All studies had the same design and demographic criteria. Following factor analysis, we further reduced the 80-item HMx to 70 items, in consultation with expert reviewers.

The 70-item HMx was then used in Adult Study 1 for initial validation, and final reduction to the short, 17-item form used in all subsequent studies. Revision of the scale to the final version included the following steps:

- Removal of items that did not load on one of the Healthy Minds Framework constructs
- Retention of items with cross-loadings below 0.30 (on orthogonal factors)
- Removal of items that cross-load on more than 2 factors (above 0.30)
- Removal of items that were the sole item to load on a factor (e.g., single-item factors)

The HMx was reduced to 58 items following the above steps, and then further reduced to the final 17-item HMx by rank ordering items based on their average correlation with well-being surveys, and then iteratively calculating alpha for each scale for the top-ranked k number of items, starting at k = 2 and incrementing by 1 until alpha reached a rounded value of 0.70 or higher. Results are reported for Adult Study 1 (and subsequent studies) with the 17 items retained in the final version.

### Validation strategy

We assessed internal consistency, convergent and divergent validity, and test-retest reliability in teens and adults in a series of 3 follow-up studies, using R statistics [16]. We used the alpha function of the psych package [17] to assess internal consistency overall, and by domain. Confirmatory and exploratory factor analysis used the *fa* function of the psych package [18–21]. Convergent validity was established for each of the four Healthy Minds framework dimensions separately, and for the entire HMx, by computing correlations for each domain with measures of similar, or overlapping, constructs in Teen Study 1 and Adult Study 1 (Table 3) using the apa.cor.table function (version 2.0.8).

**Table 3:** Measures for testing convergent and divergent validity of the Healthy Minds (HM) Index scales.

**Well-being Domain**	**Scale**	**Citation**	**Alpha** *	
			**Teen 1**	**Adult 1**
	World Health Organization Well-being Index (WHO-5)	Topp et al., 2015 [22]	0.88	0.91
	Diener Satisfaction with Life Scale (Life Sat.)	Gadermann et al., 2010 [23]	0.87	0.91
	Personal Well-being Index (PWI): Global life satisfaction	Tomyn et al., 2013 [24]	-	-
	Comprehensive Inventory of Thriving: Loneliness	Su et al., 2014 [25]	0.82	0.84
	Kessler Psychological Distress Scale (K10)	Kessler et al., 2002 [26]	0.89	0.95
Awareness	Comprehensive Inventory of Mindfulness Experiences–Adolescents (CHIME-A): Acting with awareness, Awareness of internal experiences	Johnson et al., 2017 [27]	0.73	0.85
	Emotional Styles Questionnaire (ESQ): Attention scale	Kesebir et al., 2019 [7]	0.71	0.59
	Mindful Attention Awareness Scale (MAAS) [less item 12]	Brown & Ryan, 2003 [28]	0.85	0.94
Connection	General Trust Scale	Yamagishi & Yamagishi, 1994 [29]	0.75	0.87
	Engagement, Perseverance, Optimism, Connectedness, and Happiness scale (EPOCH): Connectedness (teens)	Kern et al., 2016 [30]	0.83	-
	Positive emotion, Engagement, Relationships, Meaning and Achievement (PERMA): Relationships (adults)	Butler & Kern, 2016 [31]	-	0.86
	Dispositional Positive Emotions Scale (DPES): Compassion	Shiota et al., 2006 [32]	0.86	0.91
	Psychological Well-Being (PWB): Positive Relations	Ryff & Keyes, 1995 [2]	0.68	0.72
Insight	CHIME-A: Relativity of thoughts, Decentering and nonreactivity	Johnson et al., 2017 [27]	0.77	0.79
	Difficulties in Emotion Regulation Scale (DERS-16): Non-Acceptance of Emotion and Regulation Strategies	Gratz & Roemer, 2004 [33]	0.85	0.89
	Emotion Regulation Questionnaire (ERQ): Reappraisal (adults)	Gross & John, 2003 [34]	-	0.90
	ERQ–Children and Adolescents (CA): Reappraisal (teens)	Gullone & Taffe, 2012 [35]	0.88	-
Purpose	Francis: 1-item purpose measure	Francis, 2013 [36]	-	-
	Meaning in Life Questionnaire (Meaning)	Steger et al., 2006 [37]	0.87	0.84
	Costin: Purpose	Costin & Vignoles, 2020 [38]	0.84	0.75

*Alphas averaged if more than 1 subscale

### Transparency and openness

We report how we determined our sample size, all data exclusions, all manipulations, and all measures in the study. All data and code are available on the Open Science Framework at this url: https://osf.io/aw7bz/ (doi: 10.17605/OSF.IO/AW7BZ). This study was not preregistered.

## Results and discussion

### Scale development

We used an iterative process for assessing and revising the initial scale and individual items, which included inspecting the distribution of scores (e.g., for normalcy), inter-item correlations, and internal consistency. Below we describe how each scale was revised from the original to the final version, and the internal consistency of the final version for the scale development study samples. Cronbach’s alpha indicated very high internal consistency for each scale (Table 4), where each item (for all scales) was rated on a 1 to 5 Likert scale.

**Table 4:** Internal consistency from scale development studies.

Scale:	Awareness	Connection	Insight	Purpose
Cronbach’s Alpha	0.91	0.91	0.91	0.92
Confidence Interval	0.89, 0.92	0.90, 0.92	0.90, 0.93	0.91, 0.93

### Awareness

A large proportion of teens (15–20%, and 31% for one item) scored a 5 (“all the time”) for 6 of the 20 scale items. These items may have been subject to confirmation bias and therefore too easy to endorse. To address these issues, we changed response anchors and edited these items to make them harder. The mean awareness score was 3.40 with a standard deviation (SD) of 0.64, and mean and median inter-item correlation was 0.36, reflecting a somewhat narrow trait as intended for the dimensional approach [39].

### Connection

Participants scored near the midpoint on this 6-item subscale, with a mean of 3.7, SD of 0.60, and a minimum of 1.5. There were 5 of the 24 scale items for which no one selected option 1, or where response 5 endorsement exceeded response 4 endorsement. We determined that retaining these items would add little reliability or predictive power. Thus, we removed the corresponding items. To further support their removal, we evaluated all items based on nomological correlations. In summary, three items correlated less strongly with convergent and criterion measures than the remaining items. Two other items performed equivalently on only one measure (Engagement, Perseverance, Optimism, Connectedness, and Happiness [EPOCH]: connectedness) [30]. We interpreted these results as indicating that removing all 5 of these items would not threaten the scale’s predictive utility. All other analyses were conducted excluding these items.

### Insight

On the 22-item sub-scale, participants on average scored around the midpoint, with a mean of 3.2 and SD of 0.65. There were no items for which no one selected option 1 (out of 5), or where response 5 endorsement exceeded response 4 endorsement. We determined that no items needed to be removed.

### Purpose

On the 14-item sub-scale, participants on average scored around the midpoint, with a mean of 3.5 and SD of 0.76. There were no items for which no one selected option 1, or where response 5 endorsement exceeded response 4 endorsement. We determined that no items needed to be removed.

### Internal consistency

The revised 17-item HMIx (S1 Appendix), based on the scale development studies (described above), was used in all subsequent analyses. The HMIx showed evidence for good internal consistency, as well as moderate to good internal consistency for each of the subscales (Table 5). Visual inspection of scale histograms indicated a normal distribution of scores across the samples.

**Table 5:** Internal consistency and descriptive statistics: Healthy Minds Index.

			Teens				Adults				
Scale	Study	Mean	SD	Skew	Kurtosis	Alpha	Mean	SD	Skew	Kurtosis	Alpha
Well-being (Total score)	Study 1	3.42	0.57	-0.26	0.75	0.84	3.61	0.67	-0.45	0.76	0.92
	Study 2	3.35	0.61	-0.26	0.22	0.87	3.51	0.47	-0.26	0.22	0.83
Awareness	Study 1	3.31	0.74	-0.19	-0.04	0.60	3.64	0.72	-0.34	0.36	0.78
	Study 2	3.32	0.70	-0.19	-0.02	0.60	3.69	0.59	-0.19	-0.,02	0.72
Connection	Study 1	3.57	0.67	-0.51	0.57	0.74	3.58	0.80	-0.58	0.33	0.84
	Study 2	3.42	0.76	-0.84	1.25	0.79	3.54	0.60	-0.84	1.25	0.75
Insight	Study 1	3.20	0.81	-0.14	-0.08	0.60	3.53	0.78	-0.28	0.17	0.76
	Study 2	3.26	0.85	-0.09	-0.32	0.64	3.12	0.71	-0.09	-0.32	0.65
Purpose	Study 1	3.59	0.88	-0.51	0.09	0.84	3.71	0.80	-0.54	0.14	0.83
	Study 2	3.39	0.89	-0.66	0.45	0.83	3.69	0.81	-0.66	0.45	0.86

### Factor structure

Overall, the 4-factor structure of the HMIx was supported by the data, with the strongest evidence across exploratory and confirmatory analyses in teens and adults supporting a fit between 3 and 5 factors. All items loaded onto their corresponding dimension of the ACIP framework in the exploratory 4-factor analysis (Tables 6 and 7). The only exceptions were in the case of Awareness items 1 and 2; in the adult sample, item 1 failed to load adequately on any dimension and item 2 loaded weakly with Connection. In the teen sample, these items cross-loaded with the Insight factor (loadings = 0.35 and 0.40, on Insight, respectively; and loadings = 0.38 and 0.32 on Awareness, respectively).

**Table 6:** Factor loadings to a 4-factor solution in exploratory analysis in teens.

	Factor Number & Loading*				Within-dimension correlation
	F1	F2	F3	F4	
Awareness 1: When I want to focus, it’s easy for me.	-	-	-	0.68	0.49
Awareness 2: In general, I’m able to focus when I’m reading.	-	-	-	0.59	0.44
Awareness 3: I can notice my thoughts as soon as I have them.	-	-	0.35	0.38	0.49
Awareness 4: When some of my thoughts lead to other thoughts, I realize it while it is happening.	-	-	0.40	0.32	0.43
Connection 1: I like all of the people that I see from day to day.	-	0.43	-	-	0.44
Connection 2: I actively take time to appreciate things about the people I see from day to day.	-	0.41	-	-	0.53
Connection 3: I believe that most people are doing the best they can.	-	0.54	-	-	0.48
Connection 4: I want all people to be happy, including people I don’t like.	-	0.62	-	-	0.44
Connection 5: I care about the problems of people all over the world.	-	0.63	-	-	0.50
Connection 6: When I make decisions involving other people, I consider their best interests.	-	0.50	-	-	0.47
Insight 1: When I am interacting with someone, I reflect on how my feelings are causing me to treat them a certain way.	-	-	0.51	-	0.40
Insight 2: When I have a thought, I reflect on whether that thought is making me feel better or worse.	-	-	0.69	-	0.52
Insight 3: I can change how I feel about a situation by changing my thoughts about that situation.	-	-	0.35	-	0.35
Purpose 1: I have general life goals that make my daily activities worth doing.	0.81	-	-	-	0.72
Purpose 2: I know what’s really important in my life.	0.68	-	-	-	0.65
Purpose 3: I have a life purpose that guides my day-to-day choices.	0.77	-	-	-	0.69
Purpose 4: I know what kind of life I want to lead.	0.76	-	-	-	0.66

*Loadings > 0.30 displayed in table.

**Table 7:** Factor loadings of a 4-factor solution in exploratory analysis in adults.

	Factor Number + Loading*				Within-dimension correlation
	F1	F2	F3	F4	
Awareness 1: When I want to focus, it’s easy for me.	-	-	-	-	0.56
Awareness 2: In general, I’m able to focus when I’m reading.	-	0.32	-	-	0.53
Awareness 3: I can notice my thoughts as soon as I have them.	-	-	-	0.58	0.50
Awareness 4: When some of my thoughts lead to other thoughts, I realize it while it is happening.	-	-	-	0.62	0.43
Connection 1: I like all of the people that I see from day to day.	0.62	-	-	-	0.46
Connection 2: I actively take time to appreciate things about the people I see from day to day.	0.73	-	-	-	0.56
Connection 3: I believe that most people are doing the best they can.	0.72	-	-	-	0.48
Connection 4: I want all people to be happy, including people I don’t like.	0.65	-	-	-	0.55
Connection 5: I care about the problems of people all over the world.	0.65	-	-	-	0.46
Connection 6: When I make decisions involving other people, I consider their best interests.	0.74	-	-	-	0.47
Insight 1: When I am interacting with someone, I reflect on how my feelings are causing me to treat them a certain way.	-	-	0.46	-	0.52
Insight 2: When I have a thought, I reflect on whether that thought is making me feel better or worse.	-	-	0.71	-	0.62
Insight 3: I can change how I feel about a situation by changing my thoughts about that situation.	-	-	0.33	-	0.27
Purpose 1: I have general life goals that make my daily activities worth doing.	-	0.74	-	-	0.74
Purpose 2: I know what’s really important in my life.	-	0.62	-	-	0.66
Purpose 3: I have a life purpose that guides my day-to-day choices.	-	0.69	-	-	0.73
Purpose 4: I know what kind of life I want to lead.	-	0.62	-	-	0.74

*Loadings > 0.30 displayed in table. F = factor

Importantly, the constructs of Awareness and Insight are highly related in the ACIP Framework, and their overlap in the current validation may reflect reduced external validity of these measures as distinct, separable constructs in the general population. Since the ACIP Framework was developed as a model of the components of well-being in terms of training-based plasticity, particularly in the context of meditation and contemplative training, a critical next step is to examine their validity among meditators, and in the context of meditation training (i.e., among meditation-naïve individuals before and after meditation training). These factors are thus expected to be non-orthogonal, and we encourage researchers modelling Awareness and Insight, concurrently, to allow these factors to correlate.

Confirmatory factor analysis of the very simple structure (vss) and Velicer’s minimum average partial (MAP) supported a 2- or 3-factor solution with a maximum of 0.70 (and 0.74 in adults), and a minimum criterion of 0.09 (0.10 in adults for 2 factors), respectively. Confirmatory parallel factor analysis provided evidence for 5 factors with 3 components (with 2 components in adults). Exploratory factor analysis of a 3-factor structure in teens indicated that Insight items 2 and 3 combined with the Awareness factor, and Insight item 1 combined with the Connection factor. The exploratory analysis of the 3-factor model resulted in a Tucker Lewis Index (TLI) of 0.88, root mean square error of approximation (RMSEA) index of 0.06, and Bayesian information criterion (BIC) of -24.13, indicating an acceptable fit. Exploratory analysis of the 4-factor structure indicated a good fit (Table 6), a qualitative improvement on the 3-factor model in exploratory analysis (TLI = 0.92, RMSEA = 0.05, BIC = -174.63). See Table 8 for a summary of model fit indices for the exploratory factor analysis.

**Table 8:** Results of exploratory factor analysis: Model fits.

Sample	Factors	Tucker Lewis Index	RMSEA	Bayesian Information Criterion
Teens	3	0.88	0.06	-24.13
	4	0.92	0.05	-174.63
	5	0.95	0.04	-232.34
Adults	3	0.91	0.06	-269.93
	4	0.93	0.06	-252.02
	5	0.95	0.05	-241.79

In adults, exploratory analysis of a 3-factor structure resulted in distinct factors for Connection, Insight, and Purpose, where the Awareness items 1 and 3 loaded with Purpose, item 2 loaded with Connection, and item 4 loaded with Insight (TLI = 0.91, RMSEA = 0.06, BIC = -269.93). Exploratory analysis of the 4-factor solution in adults yielded similar results (Table 7), except Awareness items 3 and 4 loaded together on a single, distinct factor from the other domains (TLI = 0.93, RMSEA = 0.06, BIC = -252.02). Since both the 3- and 4-factor fits were acceptable in adults (rather than “good”), we also examined the 5-factor solution in an exploratory factor analysis, which produced a good fit (TLI = 0.95, RMSEA = 0.05, BIC = -241.79), whereby each factor corresponded to a distinct domain, and Awareness was split into 2 factors (items 1 and 2 loaded together, as did items 3 and 4). We additionally report the 5-factor model in teens in Table 8 for completeness.

### Convergent and divergent validity

The overall HMIx scale, as well as each of the subscales, demonstrated good convergent and divergent validity, in that each of the measures were related to measures of overall well-being (Table 9), and to similar constructs in the expected direction(s) (Table 10). The scale(s) also demonstrated good divergent validity, with relationships generally below a threshold of *r* = 0.60. The one exception with regards to divergent validity was the Purpose scale, which was consistently correlated relatively strongly with measures of similar constructs (r = 0.52 to r = 0.66).

**Table 9:** Correlations between well-being measures and the Healthy Minds Index.

Measure^+^	Study Version	Wellbeing (total)	Awareness	Connect^b^	Insight	Purpose
	(Measure mean, SD^a^)					
EPOCH^c^ (teens) / PERMA^d^ (adults)	Teen 1 (3.9, 0.9)	0.42**	0.27**	0.38**	0.21**	0.38**
	Teen 2 (3.6, 1.0)	0.48**	0.33**	0.47**	0.30**	0.37**
	Adult 1 (6.9, 2.5)	0.47**	0.37**	0.48**	0.34**	0.44**
Life Satisfaction	Teen 1 (14.7, 7.5)	0.31**	0.22**	0.25**	0.16**	0.28**
	Teen 2 (16.1, 5.0)	0.40**	0.34**	0.32**	0.19**	0.39**
	Adult 1 (22.5, 7.7)	0.41**	0.29**	0.37**	0.32**	0.43**
WHO-5^e^ Well-being Index	Teen 1 (10.9, 7.0)	0.39**	0.31**	0.29**	0.22**	0.33**
	Teen 2 (11.7, 5.7)	0.53**	0.40**	0.43**	0.30**	0.49**
	Adult 1 (14.0, 6.3)	0.49**	0.41**	0.41**	0.41**	0.46**
Distress^f^	Teen 1 (25.0, 7.3)	-0.18**	-0.29**	-0.02	0.01	-0.24**
	Teen 2 (28.0, 9.7)	-0.09	-0.12*	-0.01	0.04	-0.19**
	Adult 1 (24.5, 10.8)	-0.01	0.01	0.01	0.05	-0.08
Loneliness	Teen 1 (2.5, 1.0)	-0.29**	-0.25**	-0.17**	-0.08**	-0.31**
	Teen 2 (2.9, 1.2)	-0.28**	-0.25**	-0.16**	-0.11	-0.32**
	Adult 1 (2.7, 1.2)	-0.16**	-0.11*	-0.14**	-0.07	-0.24**

^+^See Table 3 for full names, citations, and alphas of comparison measures

^a^SD = standard deviation

^b^Connect = Connection

^c^EPOCH = EPOCH Connectedness

^d^PERMA = PERMA Relationships

^e^WHO = World Health Organization

^f^Distress = K10 Psychological Distress

**p<0.01

*p<0.05

**Table 10:** Correlations between the Healthy Minds Index scales and domain-specific measures.

		Teen Study 1			Adult Study 1		
Domain	Measure	Mean	SD^a^	Pearson’s *r*	Mean	SD^a^	Pearson’s *r*
Awareness	CHIME Act^b^	4.46	1.13	0.27**	5.04	1.34	-0.07
	CHIME Awa.^c^	4.38	1.02	0.40**	4.33	1.17	0.69**
	CHIME Dec.^d^	3.72	1.09	0.44**	4.04	1.17	0.58**
	MAAS^e^	3.25	0.81	-0.26**	3.17	1.13	0.00
	ESQ^f^ Attention	3.93	1.20	0.57**	4.61	1.15	0.41**
Connection	Trust	3.15	0.62	0.43**	3.53	0.82	0.61**
	PWB: Pos.^g^	23.96	8.85	0.32**	26.19	6.22	0.51**
	Compassion	5.49	1.06	0.59**	5.31	1.31	0.67**
Insight	CHIME Rel.^h^	4.38	0.97	0.29**	4.0	1.1	0.37**
	CHIME Dec.^i^	3.72	1.09	0.38**	4.0	1.2	0.60**
	DERS Reg.^j^	2.51	1.16	-0.01	2.6	1.2	0.01
	DERS Non-Acc.^k^	2.69	1.22	0.08**	2.6	1.3	0.04
	Reappraisal	4.60	1.21	0.40**	5.1	1.3	0.60**
Purpose	Costin	4.97	1.38	0.66**	4.87	1.35	0.52**
	Francis	3.80	1.00	0.58**	3.89	1.00	0.58**
	Meaning	4.92	1.44	0.65**	6.47	1.67	0.61**

^+^See Table 3 for full names, citations, and alphas of comparison measures

^a^SD = standard deviation

^b^Act = Acting with awareness

^c^Awa. = Awareness of internal experiences

^d^Dec. = Decentering and nonreactivity

^e^MAAS = Mindful Attention Awareness Scale

^f^ESQ = Emotional Styles Questionnaire

^g^Pos. = Positive relations with others

^h^Rel. = relativity of thoughts

^I^Dec. = Decentering

^j^Reg. = Emotion regulation

^k^Non-Acc. = Non-acceptance

**p<0.01

*p<0.05

### Test-retest reliability

The HMIx scale and subscales showed moderate to good test-retest reliability, except for Insight (Table 11). The test-retest reliability for the insight sub-scale, which ranged from an intra-class correlation (ICC) = 0.43 to 0.52, was consistently lower than the other domains (ICC range = 0.59 to 0.85, average ICC = 0.72, at a 2-week lag).

**Table 11:** Test-retest reliability: Intra-class correlations (ICC).

Construct	Teen Study 1:	Teen Study 2:	Adult Study 2:
	3-month lag	2-week lag	2-week lag
Well-Being (total)	0.65	0.75	0.81
Awareness	0.61	0.65	0.65
Connection	0.63	0.65	0.65
Insight	0.43	0.47	0.50
Purpose	0.64	0.76	0.85

### Constraints on generality

The HMIx was tested only with American participants, and primarily in online samples for the retest reliability studies. It will be important to provide evidence for the scale’s reliability and validity in diverse populations and cultures, among meditators, and from pre- to post-training in meditation.

## Conclusions

The Healthy Minds Framework was proposed by Dahl, Wilson-Mendenhall and Davidson [1] to clarify the dimensions of well-being that can be cultivated through deliberate training. In the present work, we developed a brief self-report scale that captures where people stand with regard to these dimensions. The initial evidence for the psychometric adequacy of the scale is encouraging and suggests that the Healthy Minds Index can be successfully employed to measure dimensions of well-being in both adult and teen samples. The validity of the scale as an assessment of characteristics that can change over time is important and will require additional research. In particular, evaluating responsiveness to interventions targeting the domains of well-being putatively assessed by the HMx and the predictive validity of strengthening those domains on future well-being and on the distal outcomes that are mediated by improvements in well-being is an important avenue of future research.

## Supporting information

S1 Appendix (DOCX)
