# Correction: Healthy Minds Index: A brief measure of the core dimensions of well-being

**DOI:** 10.1371/journal.pone.0345671

**Published:** 2026-03-24

**Authors:** Tammi R. A. Kral, Pelin Kesebir, Liz Redford, Cortland J. Dahl, Christine D. Wilson-Mendenhall, Matthew J. Hirshberg, Richard J. Davidson, Raquel Tatar

In the Abstract, the following information is missing after the fifth sentence of the paragraph: Additional work is needed to further refine the scale for use in adults, particularly for the awareness domain, as the confirmatory factor analysis indicated that items in that domain may reflect separable constructs.

In the Validation strategy subsection of the Methods, there is an error in the first and third sentence of the paragraph. The correct first sentence is: We assessed internal consistency, convergent and divergent validity, and test-retest reliability in teens and adults in a series of 3 follow-up studies (2 studies with teens and 1 study with adults), using R statistics (16). The correct third sentence is: Confirmatory factor analysis used the cfa function of the lavaan package (18) to model the hypothesized 4-factor structure with items in the corresponding domain, and exploratory factor analysis used the fa function of the psych package (19–22).

In the Factor structure subsection of the Results and discussion, there are multiple errors in the first paragraph. The correct paragraph is: Overall, the 4-factor structure of the HMIx was supported by the data. The strongest evidence of exploratory factor analysis in teens and adults supporting a fit between 3 and 5 factors, and confirmatory factor analysis providing evidence for the acceptable fit of a 4-factor solution in a separate sample of teens (teen study 2). All items loaded onto their corresponding dimension of the ACIP framework in the exploratory 4-factor analysis (Table 6, 7). The only exceptions were in the case of Awareness items 3 and 4, in teens, where these items cross-loaded with the Insight factor (loadings = 0.35 and 0.40, on Insight, respectively; and loadings = 0.38 and 0.32 on Awareness, respectively); in the adult sample, item 1 failed to load adequately on any dimension and item 2 loaded weakly with Purpose.

In the Factor structure subsection of the Results and discussion, there is an error in the first and second sentence of the third paragraph. The correct first sentence is: Exploratory factor analysis of the very simple structure (vss) and Velicer’s minimum average partial (MAP) supported a 2- or 3-factor solution with a maximum of 0.70 (and 0.74 in adults), and a minimum criterion of 0.09 (0.10 in adults for 2 factors), respectively. The correct second sentence is: Exploratory parallel factor analysis provided evidence for 5 factors with 3 components (with 2 components in adults).

In the Factor structure subsection of the Results and discussion, the following information is missing as the fifth paragraph: We then conducted confirmatory factor analysis of the 4-factor model with items modeled within each of the four respective domains, using independent data from the second teen study, and separately, from the second adult study. We found acceptable model fit for the 4-factor solution in teens (RMSEA = 0.058, CFI = 0.921, TLI = 0.905), though model fit criteria for adults were not in the acceptable range for the corresponding 4-factor model (RMSEA = 0.096, CFI = 0.821, TLI = 0.784). Given the results of the EFA for adult study 1, where model fit criteria were good for the 5-factor solution (and merely acceptable for 4 factors), we also tested an alternative CFA with 5 factors where awareness items were divided into two separate factors, and insight item 3 was removed. Insight item 3 loaded weakly (below 0.40) in the adult EFA, and internal consistency for the insight dimension was considerably improved for adult study 2 with removal of this item (from alpha = 0.65 to alpha = 0.77). Results of the 5-factor CFA in adults indicated improvement in model fit from the 4-factor model, though two of three model fit criteria remained just outside the acceptable range, below the 0.90 threshold. (RMSEA = 0.079, CFI = 0.891, TLI = 0.861).

In the Conclusion, the following information is missing after the third sentence of the paragraph: Further work is needed to refine the measure for use in adults, as the awareness items did not fit the expected factor structure, and may reflect separable constructs within that dimension. One insight item also loaded weakly and reduced the internal consistency when included for that dimension. Thus, scores on these dimensions in adults should be interpreted with caution.

There are errors in Table 1, 2 and 8. Please see the correct Table 1, 2 and 8 here.

**Table 1:** Summary of Healthy Minds Index validation studies in order of occurrence.

Study name	N	Objectives
Qualitative Interviews	32	Gather teens’ input on clarity of items and scale language
Scale Development (Teen Study D)	1607, total	Factor analysis & scale revision, separately for the 4 dimensions of the Healthy Minds Framework (with about n = 400, each)
Adult Study 1	420	Scale reduction, exploratory factor analysis, convergent validity and internal consistency of final 17-item scale (online; Qualtrics)
Teen Study 1	1492; 934 at retest	Exploratory factor analysis, convergent validity, internal consistency, and test-retest with 3-month interval (in-person)
Adult Study 2	281; 96 at retest	Confirmatory factor analysis, internal consistency and test-retest with 2-week interval (online; Prolific)
Teen Study 2	285; 81 at retest	Confirmatory factor analysis, convergent validity, internal consistency, and test-retest with 2-week interval (online; Qualtrics)

**Table 2:** Summary of study demographics.

Study name	Genders					Mean age, years (SD^f^)	Age Min, Max	Race & Ethnicity								
White	Black	East Asian	South Asian	Native American/ Aboriginal	Latino	Native Hawaiian/ Pacific Islander	Other	NA
										F^a^	M^b^	N^c^	O^d^	NA^e^
UX	17	3	0	0	12	16.0 (1.2)	14, 18	10	0	3	0	0	1	0	0	18
Teen D	760	817	26	4	0	16.7 (1.1)	14, 17	967	252	48	42	14	259	9	16	0
Teen 1	635	648	17	10	182	15.7 (1.2)	13, 18	441	204	53	475	11	11	63	52	182
Teen 2	159	113	10	3	0	16.0 (1.4)	14, 18	118	48	15	13	7	57	2	25	0
Adult 1	250	167	2	1	0	45.6 (17.6)	18, 85	201	70	19	9	9	84	8	20	0
Adult 2	142	137	2	0	4	45.3 (16.5)	18, 92	188	41	19	4	3	10	1	5	14

^a^F = female; ^b^M = male; ^c^N = nonbinary; ^d^O = other/ prefer not to answer; ^e^NA = no answer/ no data; ^f^SD = standard deviation

**Table 8:** Results of factor analysis: Model fits.

Study	Factors	Tucker Lewis Index	RMSEA	Bayesian Information Criterion
Teen EFA	3	0.88	0.06	−24.13
	4	0.92	0.05	−174.63
	5	0.95	0.04	−232.34
Adult EFA	3	0.91	0.06	−269.93
	4	0.93	0.06	−252.02
	5	0.95	0.05	−241.79
Teen CFA	4	0.91	0.06	13369.20
Adult CFA	4	0.78	0.10	11396.22
	5	0.86	0.08	10654.81

## References

[1] Kral TRA, Kesebir P, Redford L, Dahl CJ, Wilson-Mendenhall CD, Hirshberg MJ, et al. Healthy Minds Index: A brief measure of the core dimensions of well-being. PLoS One. 2024;19(5):e0299352. doi: 10.1371/journal.pone.0299352 38728238 PMC11086875

