# Supplementary material for: Healthy Minds Index: A brief measure of the core dimensions of well-being
Source: PLoS One. 2024 May 10;19(5):e0299352. doi: 10.1371/journal.pone.0299352 (PMC11086875; doi:10.1371/journal.pone.0299352)
Supplement: S1 Appendix — (DOCX) [file pone.0299352.s001.docx]

**Appendix**

**Healthy Minds Index**

All items are rated on a 1 to 5 Likert scale. Scores are calculated by summing the responses to each item for a given scale.

**Awareness**

Rating options 1=None of the time; 2=A little of the time; 3=Some of the time; 4=A lot of the time; 5=All of the time

- - - 1. When I want to focus, it’s easy for me.
      2. In general, I’m able to focus when I’m reading.
      3. I can notice my thoughts as soon as I have them.
      4. When some of my thoughts lead to other thoughts, I realize it while it is happening.

**Connection**

Rating options (items 1–3):1=None of the time; 2=A little of the time; 3=Some of the time; 4=A lot of the time; 5=All of the time

I like all of the people that I see from day to day.

I actively take time to appreciate things about the people I see from day to day.

I believe that most people are doing the best they can.

Rating options (items 4–6):1=Not at all; 2=A little bit; 3=Somewhat; 4=A lot; 5=To the highest degree

I want all people to be happy, including people I don’t like.

I care about the problems of people all over the world.

When I make decisions involving other people, I consider their best interests.

**Insight**
Rating options:1=None of the time; 2=A little of the time; 3=Some of the time; 4=Most of the time; 5=Every time

- - - 1. When I am interacting with someone, I reflect on how my feelings are causing me to treat them a certain way.
      2. When I have a thought, I reflect on whether that thought is making me feel better or worse.
      3. I can change how I feel about a situation by changing my thoughts about that situation.

**Purpose**
Rating options:1=Not at all; 2=A little bit; 3=Somewhat; 4=A lot; 5=To the highest degree

I have general life goals that make my daily activities worth doing.

I know what’s really important in my life.

I have a life purpose that guides my day-to-day choices.

I know what kind of life I want to lead.
